# Low dose triterpene-quinone fraction from *Ardisia crispa* root precludes chemical-induced mouse skin tumor promotion

**DOI:** 10.1186/s12906-015-0954-3

**Published:** 2015-12-05

**Authors:** Looi Ting Yeong, Roslida Abdul Hamid, Latifah Saiful Yazan, Huzwah Khaza’ai, Norhafizah Mohtarrudin

**Affiliations:** Department of Biomedical Science, Faculty of Medicine and Health Sciences, Universiti Putra Malaysia, 43400 Serdang, Selangor Malaysia; Institute of Biosciences, Universiti Putra Malaysia, 43400 Serdang, Selangor Malaysia; Department of Pathology, Faculty of Medicine and Health Sciences, Universiti Putra Malaysia, 43400 Serdang, Selangor Malaysia

**Keywords:** *Ardisia crispa*, Anti-tumor promoting, DMBA/TPA-induced skin tumorigenesis, Apoptotic index, NF-KappaB

## Abstract

**Background:**

Drastic increment of skin cancer incidence has driven natural product-based chemoprevention as a promising approach in anticancer drug development. Apart from its traditional usages against various ailments, *Ardisia crispa* (Family: Myrsinaceae) specifically its triterpene-quinone fraction (TQF) which was isolated from the root hexane extract (ACRH) was recently reported to exert antitumor promoting activity *in vitro.* This study aimed at determining chemopreventive effect of TQF against chemically-induced mouse skin tumorigenesis as well as elucidating its possible pathway(s).

**Methods:**

Mice (*n* = 10) were initiated with single dose of 7,12-dimethylbenz[α]anthracene (DMBA) (390 nmol/100 μl) followed by, a week later, repeated promotion (twice weekly; 20 weeks) with 12-*O*-tetradecanoylphorbol-13-acetate (TPA) (1.7 nmol/100 μl). TQF (10, 30 and 100 mg/kg) and curcumin (10 mg/kg; reference) were, respectively, applied topically to DMBA/TPA-induced mice 30 min before each TPA application. Upon termination, histopathological and biochemical analysis, as well as Terminal deoxynucleotidyl transferase dUTP nick end labeling (TUNEL) and transcription factor enzyme-linked immunosorbent assay (ELISA) assays were performed to elucidate the potential mechanism of TQF.

**Results:**

With comparison to the carcinogen control, results revealed that lower dose of TQF (10 mg/kg) conferred antitumor promoting effect via significant (*P* < 0.05) suppression against lipid peroxidation (LPO), apoptotic index (cell death) and nuclear factor-kappa B (NF-κB), along with reduction of keratinocyte proliferation; whilst its higher dose (100 mg/kg) was found to promote tumorigenesis by significantly (*P* < 0.05) increasing LPO and apoptotic index, in addition to aggravating keratinocyte proliferation.

**Conclusions:**

This study evidenced that TQF, particularly at its lower dosage (10 mg/kg), ameliorated DMBA/TPA-induced mouse skin tumorigenesis. Though, future investigations are warranted to determine the lowest possible therapeutic dose of TQF in subsequent *in vivo* chemopreventive studies.

## Background

Skin cancer, particularly non-melanoma skin cancers (NMSCs) which include basal cell carcinoma (BCC) and squamous cell carcinoma (SCC), appears as one of the most prevalent health concerns worldwide. Approximately 2 to 3 million cases of NMSCs and 132,000 cases of melanoma are reported globally per annum [1]. In addition to extensive exposure to sunlight and skin diseases, long term exposure to environmental or occupational carcinogens such as polycyclic aromatic hydrocarbons (PAHs) have been commonly associated with skin cancer. In this regard, chemical-induced carcinogenesis model serves vital roles in identification of novel chemopreventive agents which may target either one or plausibly multiple signaling pathways. An array of interlinking biological changes, including but not limited to epidermal hyperplasia, inflammation, proliferation and oxidative stress, are key events implicated in skin tumor promotion [2].

**Fig. 1:**
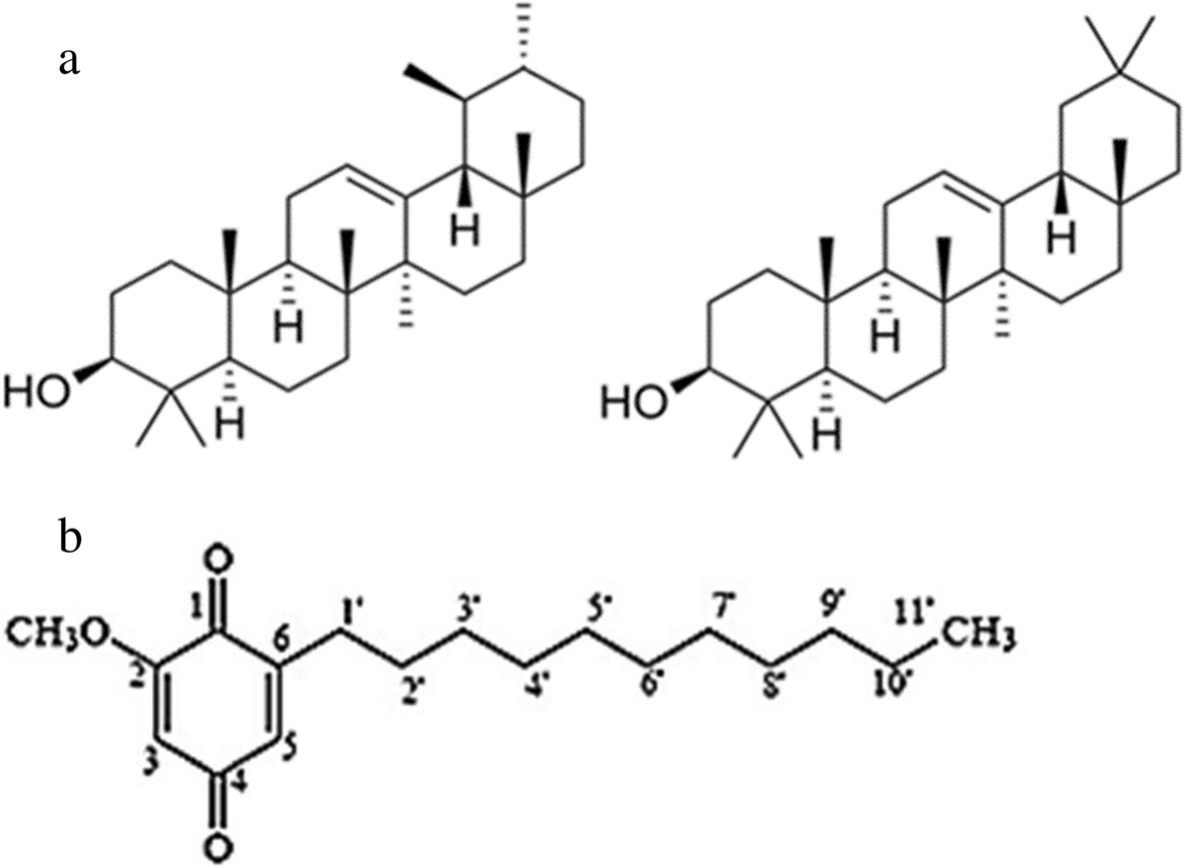
Chemical structure of (**a**) α-amyrin (left) and β-amyrin (right) exist as isomeric mixture; (**b**) 2-methoxy-6-undecyl-1,4-benzoquinone (quinone), in TQF

*Ardisia crispa* (Family: Myrsinaceae) or locally known as “*hen’s eyes”* is an evergreen shrub that has been traditionally used as treatment for rheumatism, earache, cough, fever, as well as for reduction of pain and swellings [3–5]. These traditional claims formed the basis that the plant, specifically *Ardisia crispa* root hexane (ACRH) extract, possesses anti-inflammatory, anti-hyperalgesia and anti-pyretic effects [6, 7]. In view of the close association between inflammation and cancer, ACRH and its isolated quinone-rich fraction was later proven to exhibit chemopreventive effect against mouse skin tumorigenesis [8–11]. A triterpene-quinone fraction (TQF) isolated from ACRH was also recently reported to exert antitumor promoting activity *in vitro*, whereby the effect was attributed to the synergistic action between triterpene (isomeric mixture of viminalol; Fig. 1a) and quinone compounds (2-methoxy-6-undecyl-1,4-benzoquinone; Fig. 1b) [12]*.*

Considering these, the current *in vivo* study was performed to determine chemopreventive effect of different dosages (10, 30 and 100 mg/kg) of TQF from *Ardisia crispa* roots on chemical-induced mouse skin tumor promotion. Elucidation of possible underlying mechanism(s) was focused on several molecular targets including apoptosis and biochemical/antioxidant (oxidative stress) levels [lipid peroxidation (LPO), reduced glutathione (GSH), catalase (CAT) and superoxide dismutase (SOD)] which are under control of nuclear factor-kappa B (NF-κB), activator protein-1 (AP-1) and nuclear factor-erythroid 2-related factor 2 (Nrf2).

## Methods

### Chemicals and drugs

Unless specified otherwise, all chemicals and reagents used were of analytical grade procured from Sigma-Aldrich Chemical Co. (USA).

### Plant material

Plant extraction was done on root part of *Ardisia crispa* which were obtained from Machang, Kelantan Malaysia in April, 2010. A voucher specimen (number 20841) has been authenticated by the author and deposited in the herbarium of Universiti Kebangsaan Malaysia, Bangi, Selangor.

### Extraction, separation and isolation of triterpene-quinone fraction (TQF)

TQF was isolated from ACRH and its composition was analyzed by gas chromatography (GC) with reference to compounds provided by Dr. Roslida Abd Hamid, as detailed previously [12].

### Experimental animals

Male *ICR* mice (6-8 weeks old), weighing 20-30 g, were obtained and kept at the animal house of Faculty of Medicine and Health Sciences, Universiti Putra Malaysia with ethical approval from the Animal Care and Use Committee (UPM/FPSK/PADS/BR-UUH/00315). The mice were housed 10 per cage and fed on standard laboratory diet with free access to water. Upon one week of acclimatization period, the mice were dorsally shaved with an electric hair clipper for an approximately 2 cm x 2 cm area about 1 cm off tail, three days before the commencement of experiment.

### Anti-tumor promoting effects of TQF (10, 30 and 100 mg/kg) in two-stage mouse skin tumorigenesis model

A total of 75 mice were allocated into 9 groups. Group I (vehicle control, *n* = 10) received topical application of acetone (100 μl) throughout the experimental period. Group II (carcinogen control, *n* = 10) were given a single dose of 7,12-dimethylbenz[α]anthracene (DMBA; 390 nmol/100 μl in acetone) followed by, a week later, repeated promotion with 12-*O*-tetradecanoylphorbol-13-acetate (TPA; 1.7 nmol/100 μl in acetone) twice weekly for 20 weeks. Group III (reference group, *n* = 10) was topically applied with a single dose of DMBA followed by repeated promotion with TPA twice weekly for 20 weeks (as in group II). Beginning from the ‘promotion’ period, mice were given topical application of 10 mg/kg curcumin (100 μl) 30 min before each TPA application. Groups IV, V and VI (treatment groups, *n* = 10/group) were treated as in group III, with the only difference that 10 mg/kg TQF (group IV), 30 mg/kg TQF (group V) and 100 mg/kg TQF (group VI), instead of 10 mg/kg curcumin, were applied to the mouse skin; whilst groups VII, VIII and IX (treatment control groups, *n* = 5/group) received only 10 mg/kg TQF (group VII), 30 mg/kg TQF (group VIII) and 100 mg/kg TQF (group IX) for 20 weeks, with neither DMBA nor TPA application.

### Morphological assessment

Along the experimental period, body weight of mice and tumor size (length, width and height; measured with a vernier caliper) were observed and measured at weekly interval. Only tumors that persisted for more than one week with diameter greater than 1 mm were taken into consideration for data analysis. The following parameters were evaluated: (i) latency period of tumor formation was determined when the first tumor appeared; (ii) percentage of tumor incidence was calculated by dividing the number of tumor-bearing mice with the total number of mice in a group and multiplied with 100 %; (iii) tumor burden was obtained by dividing the total number of tumors with the number of tumor-bearing mice in a group; (iv) tumor volume was measured by multiplying Π/6 to the length, width and height of tumor.

### Sample preparation

At the end of week 20, mice were sacrificed by cervical dislocation and sampled for further analysis. Treated skin area (with or without tumor) and liver were harvested. Part of the harvested skin was preserved in 10 % (v/v) neutral buffered formalin for histopathological analysis and *in situ* cell death detection; whilst, the remaining was kept in liquid nitrogen for protein expression analysis. Harvested liver was cleaned with normal saline and stored in liquid nitrogen for biochemical analysis.

### Histopathological analysis

Formalin-fixed, paraffin-embedded mouse skin samples were processed and stained according to routine Haematoxylin and Eosin (H&E) protocol. Stained slides were observed under light microscope and digital micrographs of the slides were taken.

### *In situ* cell death detection

*In situ* cell death detection was performed according to manufacturer’s protocol of terminal deoxynucleotidyl transferase (TdT)-mediated deoxyuridine triphosphate (dUTP) nick end labeling (TUNEL), peroxidase (POD) assay kit from Roche Molecular Biochemicals (Mannheim, Germany). Formalin-fixed, paraffin-embedded skin samples were sectioned (4 μm) onto silanized slides which were deparaffinized, followed by incubation in freshly prepared permeabilization solution (0.1 % Triton X-100 in 0.1 % sodium citrate). TUNEL reaction mixture was then added to the sample. Next, the sample was exposed to converter-POD, DAB substrate and finally counterstained with Haematoxylin. TUNEL positive cells (apoptotic cells) were counted from 10 randomly chosen fields at 400-fold magnification and expressed as apoptotic index by using the formula: number of apoptotic cells*100/total number of counted cells.

### Biochemical analysis

Liver samples were prepared as 10 % (w/v) tissue homogenate in 0.15 M Tris-HCl (pH 7.4) [13]. Part of the homogenate was used for estimation of LPO [14] and GSH [15]; whilst the remaining homogenate was centrifuged at 10,000 *g* (4 °C) for 15 min and the supernatant was used for determination of SOD [16], CAT [17] and protein estimation [18]. Triplicate measurements were made for each liver sample.

### Protein expression of transcription factors via enzyme-linked immunosorbent assay (ELISA)

Monoclonal antibodies for AP-1/total c-Jun, NF-κB/p65 and Nrf2, as well as horseradish peroxidase (HRP)-conjugated goat anti-rabbit IgG secondary antibody were purchased from Cell Signaling Technology (USA). 10 % skin homogenate was prepared in 50 mM Tris–HCl (pH 7.4) under ice-cold condition. 100 μl of the sample supernatant (2 μg protein/ml in carbonate-bicarbonate buffer, pH 9.6) was loaded in triplicates into a 96-well ELISA plate and kept overnight at 4 °C. The plate was then washed and blocked with 300 μl bovine serum albumin (5 %, w/v) before incubation for 1 h at 37 °C. After washing, 100 μl of diluted primary antibody (NF-κB, c-Jun or Nrf2; 1:1000 in blocking buffer) was added to each well and incubated for 2 h at 37 °C before addition of 100 μl of diluted secondary antibody (1:1000 in blocking buffer). After another 2 h of incubation at 37 °C, 100 μl 2,2’-azino-bis(3-ethylbenzothazoline-6-sulfonic acid) (ABTS; 1 mM) substrate containing 1 μl hydrogen peroxide (H_2_O_2_; 30 %) per ml of ABTS solution was added into each well. Color was allowed to develop for 20 min in the dark and the plate was read immediately at 405 nm.

### Statistical analysis

Data were expressed as mean ± standard error of mean (S.E.M.). Statistical analysis was performed with SPSS 20.0 by using one-way analysis of variance followed by Least Significant Difference (LSD) post hoc test to compare means between groups. Values with *P* < 0.05 were considered statistically significant.

## Results

### Antitumor promoting effects of TQF (10, 30 and 100 mg/kg) in two-stage mouse skin tumorigenesis model

Protective effect of topical application of TQF (10, 30 and 100 mg/kg) in DMBA/TPA-induced mouse skin tumors during the ‘promotion’ stage was evaluated and the findings were summarized in Table 1. It is prominent that topical application of TQF for 20 weeks in mice did not result in remarkable difference in body weight among all groups. There was also an absence of observable tumor development in group I (vehicle control) and groups VII-IX (treatment control). Though, mice in the carcinogen control group (group II) exhibited tumor development beginning from week 9 (data not shown). A distinguishing delay in tumor formation was noted in mice treated with 30 mg/kg TQF (group V, week 11) and 10 mg/kg TQF (group IV, week 14). Surprisingly, increasing TQF dosage seems to promote tumor development in which tumor was documented as early as week 6 in mice treated with 100 mg/kg TQF (group VI).

**Table 1:** Effects of TQF (10 mg/kg, 30 mg/kg and 100 mg/kg) on ‘promotion’ stage of DMBA/TPA-induced mouse skin tumorigenesis after 20 weeks

Group	Body weight (g)	Tumor Incidence (%)	Tumor burden	Tumor volume (mm^3^)	
	Initial	Final			
I	20.5 ± 0.43	32.4 ± 0.93	-	-	-
II	23.3 ± 0.54	35.4 ± 1.01	60^a^	2.00 ± 0.37^a^	7.62 ± 3.51^a^
III	23.6 ± 0.64	32.3 ± 0.56	25^b^	1.50 ± 0.50^a^	4.59 ± 3.58^a,b^
IV	20.7 ± 0.50	32.8 ± 0.97	33.3^c^	1.00 ± 0.00^b^	1.48 ± 0.37^b^
V	21.8 ± 0.49	33.3 ± 1.09	77.8^a^	2.29 ± 0.52^a^	9.16 ± 3.21^c^
VI	21.7 ± 0.30	31.6 ± 0.70	100^d^	7.30 ± 1.07^c^	13.67 ± 5.29^d^
VII	22.4 ± 0.81	33.2 ± 1.98	-	-	-
VIII	21.2 ± 0.73	32.8 ± 0.49	-	-	-
IX	20 ± 0.48	33.8 ± 1.18	-	-	-

Values expressed as mean ± S.E.M. (group I-VI, *n* = 10; group VII-IX, *n* = 5)

Values with different superscript letters(^a,^ ^b,^ ^c,^ ^d^) within the same column are statistically different (*P* < 0.05)

Group: I (vehicle control); II (carcinogen control); III (reference group, 10 mg/kg curcumin); IV (10 mg/kg TQF); V (30 mg/kg TQF); VI (100 mg/kg TQF); VII (treatment control, 10 mg/kg TQF); VIII (treatment control, 30 mg/kg TQF); IX (treatment control, 100 mg/kg TQF)

We also observed that, at the end of the study, group IV (33.3 %) exhibited significantly lower (*P* < 0.05) percentage of tumor incidence than group II (60 %); whilst the values escalated in mice treated with higher dosages of TQF, accounting for 77.8 % and 100 % tumor incidence in groups V and VI, respectively. As the reference compound, curcumin-treated group (group III) showed only 25 % tumor incidence. Group IV (1.00 ± 0.00) also showed significantly lower (*P* < 0.05) tumor burden than group II (2.00 ± 0.37) and group III (1.50 ± 0.50); whilst its tumor volume (1.48 ± 0.37 mm^3^) was comparable to that of group III (4.59 ± 3.58 mm^3^), but at significantly lower value (*P* < 0.05) than group II (7.62 ± 3.51 mm^3^).

### Histopathological analysis

**Fig. 2:**
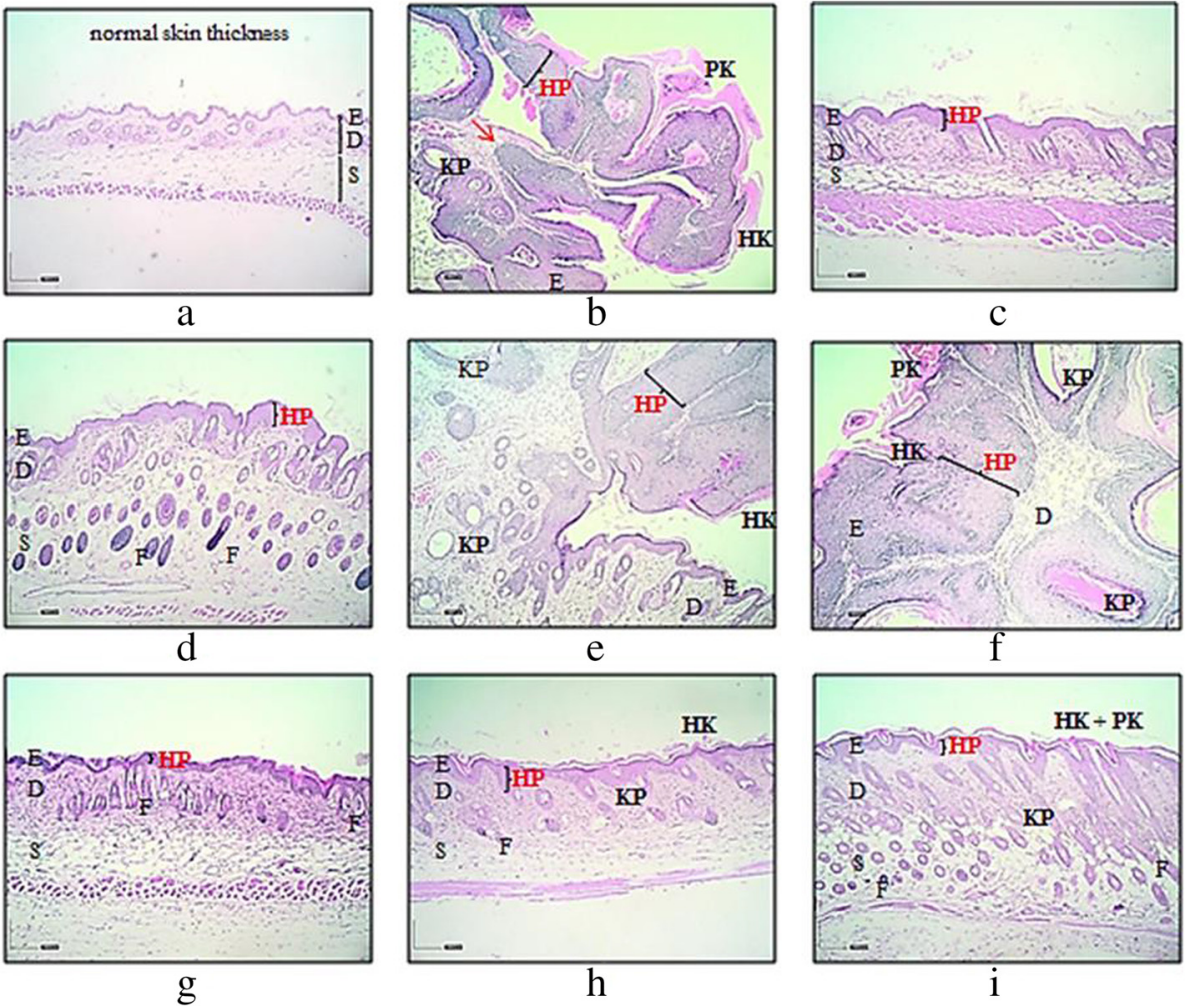
Representative microphotographs of H&E sections (original magnification, x40) from mice skin. (**a**) acetone (group I; vehicle control); (**b**) DMBA/TPA (group II; carcinogen control); (**c**) curcumin (group III; reference group); (**d**) 10 mg/kg TQF (group IV; treatment group); (**e**) 30 mg/kg TQF (group V; treatment group); (**f**) 100 mg/kg TQF (group VI; treatment group); (**g**) 10 mg/kg TQF (group VII; treatment control); (**h**) 30 mg/kg TQF (group VIII; treatment control) and (**i**) 100 mg/kg TQF (group IX; treatment control). Note: (E) epidermis; (D) dermis; (S) subcutaneous tissue; (F) hair follicle; (HP) hyperplasia; (HK) hyperkeratosis; (PK) parakeratosis; (KP) keratin pearls. Arrow (in red) indicates papillomatous growth with intact basement membrane

Microscopic examination of treated skin, with or without tumor formation, after 20 weeks of promotion period showed presence of hyperplasia, hyperkeratosis and/or parakeratosis, except for group I (Fig. 2). Tumors formed showed papillomatous growth of benign characteristic with well-bordered basement membrane and no evidence of invasion to indicate malignant changes. As carcinogen control, group II showed hyperplastic epidermal layer along with hyperkeratosis, parakeratosis and mild increment in the number of keratin pearls. Group III, though, exhibited only mild epidermal hyperplasia. Among TQF-treatment groups (groups IV-VI), the least hyperplastic epidermal layer was observed in group IV, followed by groups V and VI. Hyperkeratosis and parakeratosis were also observed in group VI; whilst group V displayed only mild hyperkeratosis and group IV showed an absence of both features. Increased formation of keratin pearls was also noted in both groups V and VI. Mild hyperplasia were observed in all treatment control groups (groups VII-IX), in addition to mild hyperkeratosis and mild increment in the amount of keratin pearls in group VIII, as well as mild hyperkeratosis, parakeratosis and severe increment in the number of keratin pearls in group IX.

### *In situ* cell death detection

**Fig. 3:**
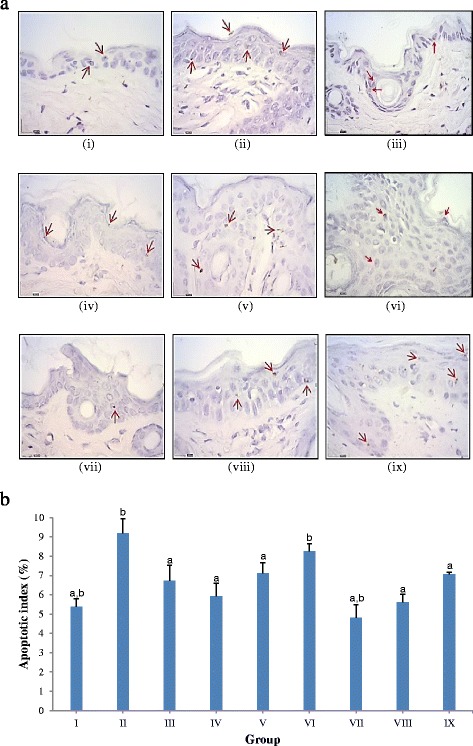
Effects of TQF (10 mg/kg, 30 mg/kg and 100 mg/kg) on apoptosis. (**a**) Apoptotic index. Values expressed as mean ± S.E.M. (group I-VI, *n* = 10; group VII-IX, *n* = 5). ^a^ *P* < 0.05 compared with carcinogen control; ^b^ *P* < 0.05 compared with curcumin; (**b**) Representative microphotographs of TUNEL staining (original magnification, ×400) for apoptosis in mice skin during ‘promotion’ stage of DMBA/TPA-induced tumorigenesis. (i) group I (vehicle control); (ii) group II (carcinogen control); (iii) group III (reference group, 10 mg/kg curcumin); (iv) group IV (10 mg/kg TQF); (v) group V (30 mg/kg TQF); (vi) group VI (100 mg/kg TQF); (vii) group VII (treatment control, 10 mg/kg TQF); (viii) group VIII (treatment control, 30 mg/kg TQF); (ix) group IX (treatment control, 100 mg/kg TQF). Arrows (in red) indicate brownish-stained apoptotic cells

Apoptotic effect of TQF on mouse skin tumor promotion was determined by using TUNEL assay, as depicted in Fig. 3a and Fig. 3b (representative micrographs). Upon stimulation by carcinogens, we noticed an increase in apoptotic index in group II (9.19 ± 0.76 %) in comparison to group I (5.39 ± 0.40 %). Conversely, group III (6.72 ± 0.80 %) showed significantly reduced apoptotic index (*P* < 0.05), whereas an approximate reduction of 1.6- and 1.3-fold were observed in group IV (5.92 ± 0.69 %) and group V (7.11 ± 0.55 %) respectively, with respect to group II. Yet, an inconsequential to group II apoptotic index was noted in group VI (8.26 ± 0.39 %). It was also interesting to note that treatment with TQF alone, without DMBA/TPA induction, increased apoptosis dose-dependently. Such findings infer a dual role of TQF in modulating apoptotic effect in which lower doses of TQF (10 mg/kg and 30 mg/kg) provided safeguard against DMBA/TPA-induced apoptosis; whereas higher dose of TQF (100 mg/kg) was found to potently mediate DNA damage, even though in the absence of carcinogens.

### Biochemical analysis

In comparison to the carcinogen control [group II, 1.82 ± 0.09 nmol malondialdehyde (MDA)/g tissue; Table 2], we found that topical application of 10 mg/kg TQF (group IV, 1.37 ± 0.16 nmol MDA/g tissue) significantly reduced (*P* < 0.05) MDA production to a level that was comparable to that of group III (1.30 ± 0.23 nmol MDA/g tissue). Though, prominent deterioration of LPO was demonstrated by mice in group VI (2.26 ± 0.19 nmol MDA/g tissue). A similar trend was also observed in the treatment control groups (groups VII-IX).

**Table 2:** Biochemical analysis (LPO, GSH, CAT and SOD)

Group	LPO (nmol MDA/g tissue)	GSH (μg/mg tissue)	CAT (U/μg protein)	SOD (U/mg protein)
I	1.65 ± 0.10	0.84 ± 0.05^a^	1.97 ± 0.13^b^	4.93 ± 0.42
II	1.82 ± 0.09^b^	0.61 ± 0.06^b^	2.33 ± 0.14^b^	4.47 ± 0.41
III	1.30 ± 0.23^a^	0.95 ± 0.07^a^	0.81 ± 0.06^a^	4.98 ± 0.39
IV	1.37 ± 0.16^a^	0.77 ± 0.09	1.67 ± 0.33^a,b^	5.35 ± 0.45
V	1.48 ± 0.19	0.89 ± 0.09^a^	0.83 ± 0.07^a^	5.82 ± 0.38
VI	2.26 ± 0.19^a,b^	0.95 ± 0.07^a^	0.95 ± 0.06^a^	4.78 ± 0.25
VII	1.35 ± 0.16	0.75 ± 0.10	0.86 ± 0.10^a^	4.71 ± 0.50
VIII	1.76 ± 0.13	0.66 ± 0.11^b^	0.64 ± 0.06^a^	4.49 ± 0.43
IX	2.14 ± 0.17^b^	0.45 ± 0.07^b^	0.84 ± 0.07^a^	5.84 ± 0.58

^a^ *P* < 0.05 compared with carcinogen control; ^b^ *P* < 0.05 compared with curcumin

On the other hand, considerable depletion of GSH was noted in group II (0.61 ± 0.06 μg/mg liver tissue), in relative to group I (0.84 ± 0.05 μg/mg liver tissue). Pretreatment with TQF on DMBA/TPA-induced mice restored the depletion in dose-dependent manner, reporting 0.77, 0.89 and 0.95 μg/mg liver tissue for groups IV, V and VI respectively, with insignificant difference among the groups. Such protective effect by TQF was comparable to those exerted by curcumin (group III, 0.95 ± 0.07 μg/mg liver tissue). Though, treatment with TQF alone seems to exert toxicity in mice wherein depleting GSH activities were observed with parallel increment of TQF dosages.

Yet, astonishingly, high level of CAT was found in group II (2.33 ± 0.14 U/μg protein). This could be an adaptive response of the mice to increasing oxidative stress induced by DMBA/TPA application. The adaptive response was apparently reduced with topical application of TQF (groups IV-IX) and curcumin (group III), as indicated by significant reduction of CAT activity (*P* < 0.05) in these groups. We also observed an overall absence of significant difference between groups in SOD activity which is indicative of an inconsequential protective role of such enzyme in mediating antitumor promoting activity of TQF.

### Protein expression of transcription factor via ELISA

**Fig. 4:**
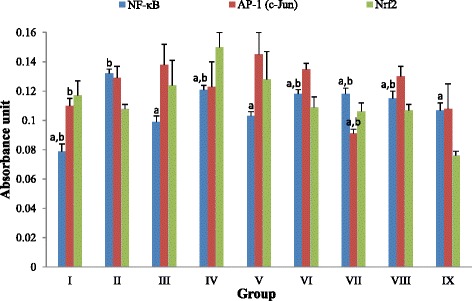
Effects of TQF (10 mg/kg, 30 mg/kg and 100 mg/kg) on protein expression of NF-κB, AP-1 (c-Jun) and Nrf2. ^a^ *P* < 0.05 compared with carcinogen control; ^b^ *P* < 0.05 compared with curcumin. Values expressed as mean ± S.E.M. (group I-VI, *n* = 10; group VII-IX, *n* = 5). Values with different superscript letters within the same column are statistically different (*P* < 0.05). Group: I (vehicle control); II (carcinogen control); III (reference group, 10 mg/kg curcumin); IV (10 mg/kg TQF); V (30 mg/kg TQF); VI (100 mg/kg TQF); VII (treatment control, 10 mg/kg TQF); VIII (treatment control, 30 mg/kg TQF); IX (treatment control, 100 mg/kg TQF)

Our results (Fig. 4) on the possible involvement of transcription factors (NF-κB, AP-1/c-jun, Nrf2) in mediating antitumor promoting effect of TQF showed that TQF inhibited tumor promotion in mouse skin by suppressing NF-κB. This was indicated by marked reduction of absorbance in NF-κB level in groups IV-IX in comparison to group II (0.132 ± 0.003). The most noteworthy reduction of NF-κB expression was seen in group V (0.103 ± 0.003), at comparable level to that of group III (0.099 ± 0.004). As a transcription factor that mainly governs cellular proliferation, AP-1/c-Jun protein expression was augmented in group II (0.129 ± 0.008). The expression was also raised with topical application of TQF (groups IV-VI) and curcumin (group III) at insignificant difference with respect to group II. Such findings imply the restricted impact of those treatments on AP-1 protein, viz. the treatments caused neither further proliferation nor inhibition towards mouse skin tumors development. On the other hand, there was an overall insignificant difference of Nrf2 protein expression level between groups. Though the difference is not statistically significant, decreased expressions were noted in group II (0.108 ± 0.003) and group VI (0.109 ± 0.007) when compared to group I (0.117 ± 0.010). In comparison to group II, the Nrf2 protein level was increased upon treatment with 30 mg/kg TQF (group V, 0.128 ± 0.019) and was further augmented when 10 mg/kg TQF (group IV, 0.150 ± 0.010) was applied to DMBA/TPA-induced mice, thus suggesting a promising role of TQF in up regulating Nrf2 expression at low dosages.

## Discussion

Chemoprevention employs naturally occurring or synthetic chemical agents to block, suppress or reverse carcinogenesis at an early stage before invasion or metastasis occur [19, 20]. In comparison to the initiation stage which is an irreversible event, the promotion stage occurs over an extended period of time that may be reversible during the course of tumor development [21] Thus, prevention of tumor promotion is customarily regarded as a more efficient approach in cancer chemoprevention. In recent years, research has shifted towards developing novel chemopreventive agent from natural products, especially plants. For they represent a vast vault of phytochemicals with diverse medicinal usages that have been exploited since the ancient times, as seen in traditional medicines such as Ayurveda (India) and Acupuncture (China).

Years of inflammatory-related research have led to surprising discovery that *Ardisia cirspa* indeed possesses potential antitumor property, despite a lack of understanding on the possible mechanism(s) involved [8–11]. In our present effort to explore, for the very first time, the mechanism(s) underlying antitumor property of *Ardisia crispa*, focus has been put on exploring its effect of cellular apoptosis, biochemical/antioxidant markers (oxidative stress) levels and the associated transcription factors (NF-κB, AP-1 and Nrf2). Reactive oxygen species (ROS) are highly indicative as one of many causative factors in tumour promotion whereas antioxidants such as GSH, SOD and CAT are indispensable for protection against the deteriorating effects.

In response to repetitive treatment with carcinogen, sustained epidermal hyperplasia or cell proliferation occurs preceded by inflammation and followed by formation of papillomas that may eventually lead to SCC [21, 22]. Though, a large proportion of tumors formed over a period of 20 weeks are merely papillomas that rarely progress into SCC [22, 23]. Shishodia et al. documented that activation of NF-κB upon DMBA/TPA stimulation is closely associated with increased keratinocyte proliferation [24]. From our study, we found that TQF suppressed tumor promotion at lower dosage (10 mg/kg) but potentiated tumor formation with increasing dosages (30 mg/kg and 100 mg/kg) in DMBA/TPA-induced mice. We postulate that 10 mg/kg TQF modulated cellular proliferation in similar manner as curcumin did by causing profound inhibition against NF-κB-regulated cyclin D1, as evidenced by their significant suppression on NF-κB (Fig. 4) [24]. Nevertheless, study has also reported that tumor necrosis factor (TNF) receptor-associated factor 2 (TRAF2) may interact with either TRAF for NF-κB (TANK) or mitogen-activated protein kinase kinase kinase (MEKK1). Thus, instead of interacting with TANK, we further postulate that higher dosage of TQF could have interact with the latter (MEKK1) and caused activation of AP-1, as well as subsequent cell proliferation [25].

We also investigated on the effect of TQF on cellular apoptosis. Application of tumor promoter has been reported to augment cellular ROS levels and cause deoxyribonucleic acid (DNA) double-strand breaks, which may ultimately lead to apoptosis if cells suffer from extensive DNA damage [26–28]. In this regard, increased apoptotic index (Fig. 3a) upon application of DMBA/TPA in the current study is strongly believed to arise from carcinogen-induced oxidative stress, as exemplified by a significant increase in LPO level (Table 2). Intervention with curcumin caused noticeable protective role against DNA damage-induced apoptosis by reducing oxidative stress (significant diminution of LPO and up regulation of GSH), as previously reported [29]. These effects were mediated via down regulation of oxidant-generating genes such as NF-κB [30]. Considering that, it is reasonable to deduce that 10 mg/kg and 30 mg/kg TQF mediated protection against apoptosis via similar manner. i.e reduction of oxidative stress (lowered LPO and increased GSH) (Table 2) and suppression of NF-κB (Fig. 4).

On the contrary, increasing TQF dosage (100 mg/kg) seems to confer toxic effects attributable to the increasing amount of quinone compound present. Aside from its vital biological function, as in endogenous biomolecules such as phylloquinone (vitamin K) and ubiquinone (coenzyme Q10), quinone is generally cytotoxic and/or genotoxic. Quinones are Michael acceptors that are capable of alkylating effectively at nucleophilic sites on macromolecules such as DNA or proteins. They are also found to react spontaneously with GSH to produce GS-hydroquinones that are readily autoxidized to form semiquinones, superoxide anion (O_2_^•−^) and H_2_O_2_ [31]. This reaction not only depletes cellular GSH levels, but also leads to increased production of ROS which are both devastating factors to cells. Alternatively, quinones are redox active compounds that are prone to reductive activation. An electron is transferred from reduced nicotinamide adenine dinucleotide phosphate (NADPH)-cytochrome P450 reductase to quinone, leading to production of semiquinone which are readily oxidized back to the parent quinone, in the presence of oxygen, via reduction of oxygen to O_2_^•−^ [32]. This redox cycling leads to massive production of O_2_^•−^ and eventual generation of oxidative stress condition.

LPO is a renowned marker of oxidative stress as it is known to increase production of epoxides, hydroperoxides, and MDA that may interact with protein and DNA, thus leading to tumor formation. Ursolic acid, a type of triterpene compound that shows close structural resemblance to the triterpene compounds (amyrins) under study, was evidenced to protect human lymphocytes against ultraviolet B (UVB)-induced oxidative stress through its significant reductive effect on LPO [33]. This suggests that the presence of amyrins in TQF may, at least in part, confer similar protective effect. Meanwhile, antioxidants, both non-enzymatic (GSH) and enzymatic (SOD and CAT), are reputable for protection against oxidative stress. Our findings indicate that pretreatment with curcumin and TQF not only restored the depleted GSH levels (Table 2), but might have also induced GSH synthesis. This is supported by Dickinson et al. and Biswas et al. who reported that curcumin was capable of increasing expression of both catalytic and modifier subunits of glutamate cysteine ligase (GCL) that are needed for GSH biosynthesis [34, 35] Whilst, reduction of GSH level in mice treated with TQF alone (without DMBA/TPA application) as a result of TQF-induced pro-oxidant environment that causes oxidation of GSH to glutathione disulfide (GSSG) is highly indicative [36].

Although diminished levels of antioxidants are frequently implicated under oxidative stress condition, exceptions to the widely accepted statement have been previously reported. Rats exposed to acute intoxication with chlorfenvinphos were reported to significantly increase hepatic CAT activity, as compared to the control (untreated) group. It was explicated that the increased CAT level was due to response of the liver to high levels of H_2_O_2_ [37]. Such an increased CAT activity is in agreement with the current findings in DMBA/TPA-induced mice (Table 2). Nonetheless, it is supremely important to note that elimination of H_2_O_2_ does not occur solely via CAT activity, for GPx also possesses essential role in exerting similar effect. Both GPx and CAT are known to act under different conditions of H_2_O_2_ wherein low concentration of H_2_O_2_ is principally acted upon by GPx whereas CAT is indispensable when GPx reaches saturation in the presence of high amount of H_2_O_2_ [38]. In this regard, curcumin and TQF (30 mg/kg and 100 mg/kg) are postulated to cause greater inhibition against H_2_O_2_ production than that in the vehicle control along with prominent up regulation of GPx expression to an extent that prevailed the amount of H_2_O_2_. This is parallel to previous findings by Sevgiler et al. whom reported that curcumin increased GPx levels while concomitantly lowered CAT activity in response to cadmium-induced oxidative stress [39]. Moreover, neither application of DMBA/TPA, curcumin nor TQF in the current study resulted in significant change in SOD levels as compared to the vehicle control. Similar findings have been reported by Guangwei et al. who found insignificant change in SOD levels in rats intoxicated with acrylonitrile and those pretreated with curcumin [40].

Transcription factors, particularly NF-κB, AP-1 and Nrf2, are signaling molecules with vital roles in mediating inflammatory response, cellular proliferation and either antioxidative or detoxification systems. From our analyses, it is ubiquitous that application of DMBA/TPA on mouse skin resulted in activation of NF-κB, as similarly reported previously [41]. As a transcription factor that plays major role in inflammation, down regulation of NF-κB conferred by curcumin or TQF as a consequence of their anti-inflammatory action is highly suggestive. Indeed, curcumin was shown to down regulate NF-κB via inhibition of IκBα phosphorylation, IκBα degradation, p65 phosphorylation and p65 nuclear translocation which subsequently correlated with down regulation of downstream cyclooxegenase-2 (COX-2) expression [42] Likewise, TQF which contains COX-2 inhibitor (quinone compound) is also supposed to act through similar mechanism of NF-κB suppression [43]. Such postulation is based on the presence of a functional group (phenyl methoxy group) common to both agents. Curcumin possesses two phenyl methoxy groups (-OCH_3_) in its chemical structure and it was found to confer the most active suppressive effect against NF-κB activation. The extent of inhibition diminished when demethoxycurcumin (one phenyl methoxy group) was applied and the least suppression was exhibited by bisdemethoxycurcumin (no phenyl methoxy group). These findings infer the importance of phenyl methoxy moiety, which is essentially present in the quinone compound (Fig. 1b) of TQF, at suppressing NF-κB activation albeit a lack of understanding in the reaction(s) involved [44].

Although simultaneous inhibition of AP-1/c-Jun along with down regulation of NF-κB is commonly exhibited by chemopreventive agents including curcumin, this is not always the case. Dickinson et al. reported that the total c-Jun content (phosphor- and non-phosphorylated) did not change significantly upon exposure to curcumin, but a dramatic two-fold increase in phosphorylated (activated) c-Jun was noted [34]. As revealed by Sabapathy et al., different c-Jun N-terminal kinases (JNKs) isoforms act antagonistically in mediating c-Jun expression. JNK1 is more efficient in phosphorylating c-Jun whilst JNK2 appears to target c-Jun for degradation [45]. Under such circumstances, curcumin and TQF are postulated to stimulate activation of both JNKs isoforms, leading to an increased c-Jun phosphorylation along with concomitant depletion of c-Jun protein and thus, resulted in insignificantly modulated total c-Jun content (Fig. 4).

Nrf2 is another transcription factor that is inevitably needed in preventing oncogenic response via regulation of antioxidants or detoxifying genes. Down regulation of endogenous Nrf2 expression has been implicated upon DMBA/TPA treatment in mice skin [46]. Curcumin, as well as triterpenoid and quinone compounds, contains α,β-unsaturated carbonyl moiety that is capable of interacting with thiol group (-SH) in Kelch-like ECH-associated protein 1 (Keap1) via Michael addition reaction [47–49]. This dissociates Nrf2 from Keap1-Nrf2 complex, saving it from ubiquitin-target degradation and thus, increases its level. Whilst low concentration of Michael acceptor binds preferentially to Keap1, high concentration of the agent was found to target and inhibit proteins with lower binding affinity [50]. Under such condition, Nrf2-Keap1 complex remains uninterrupted and Nrf2 is destabilized through rapid degradation which resulted in its reduced detectable levels, as exhibited by mice treated with 100 mg/kg TQF (Fig. 4).

## Conclusions

Compiling findings from the present study, it is noteworthy that *in vivo* antitumor promoting activity of TQF was restricted by dosage whereby lower dose of TQF (10 mg/kg) suppressed DMBA/TPA-induced mice skin papilloma; whilst higher dose of TQF (100 mg/kg) was found to potentiate tumor development. This was further reinforced by mechanism studies in which the lower dose (10 mg/kg) was shown to confer antitumor promoting effect via significant (*P* < 0.05) suppression against LPO, apoptotic index (DNA damage) and NF-κB, along with reduction of keratinocyte proliferation. Based on these results, it is suggested that TQF be examined at much lower doses in subsequent *in vivo* tumorigenesis study until the lowest possible therapeutic dose is attained.
